# Obituary Notices

**Published:** 1847-04

**Authors:** 


### ARTICLE XII.

### OBITUARY.

Dr. John Thompson, late Professor of General Pathology in the University of Edinburg, died on the 11th of October last, aged 82 years. He was highly distinguished both as a teacher, writer, and practitioner.

Dr. Bostock, a highly distinguished Physician, of London, died in August last, at the age of 73 years, of cholera.

Dr. Lyman Brackett, of Rochester, Ind., a former contributor to our Journal, we learn by a letter from a friend, died on the 7th of April, inst. His disease was not reported.

Dr. N. Worcester, of Cincinnati, Ohio, highly distinguished as a teacher and writer, died recently of acute bronchitis.

Prof. Tommasini, of Italy, author of the contra-stimulant doctrine in medicine, died some time since.

Also, Augustus Berard, Prof. of Clinical Surgery in Paris.

Also, December 17th, M. Brouissonnet, Prof. of Clinical Medicine in the Faculty of Montpelier, aged 80 years.

Also, on 4th February, M. Dutrochet, of Paris, the discoverer of the property of endosmosis and exosmosis in organic substances, in the 70th year of his age.

